# Disturbances of Hormonal Circadian Rhythms by Light Pollution

**DOI:** 10.3390/ijms24087255

**Published:** 2023-04-14

**Authors:** Michal Zeman, Monika Okuliarova, Valentina Sophia Rumanova

**Affiliations:** Department of Animal Physiology and Ethology, Faculty of Natural Sciences, Comenius University, 842 15 Bratislava, Slovakia; monika.okuliarova@uniba.sk (M.O.); rumanovavalentina@gmail.com (V.S.R.)

**Keywords:** artificial light at night, neuroendocrine system, vasopressin, melatonin, corticosterone, testosterone, thyroid hormones

## Abstract

The circadian rhythms evolved to anticipate and cope with cyclic changes in environmental conditions. This adaptive function is currently compromised by increasing levels of artificial light at night (ALAN), which can represent a risk for the development of diseases of civilisation. The causal links are not completely understood, and this featured review focuses on the chronodisruption of the neuroendocrine control of physiology and behaviour by dim ALAN. The published data indicate that low levels of ALAN (2–5 lux) can attenuate the molecular mechanisms generating circadian rhythms in the central oscillator, eliminate the rhythmic changes in dominant hormonal signals, such as melatonin, testosterone and vasopressin, and interfere with the circadian rhythm of the dominant glucocorticoid corticosterone in rodents. These changes are associated with a disturbed daily pattern of metabolic changes and behavioural rhythms in activity and food and water intake. The increasing levels of ALAN require the identification of the pathways mediating possible negative consequences on health to design effective mitigation strategies to eliminate or minimise the effects of light pollution.

## 1. Introduction

Most organisms, including humans, live in an environment that exhibits cyclic changes with different periods. The most prominent are daily cycles caused by the rotation of the Earth around its axis. To adapt to these day/night changes, organisms developed internal circadian (circa—about; dies—day) clocks to adjust physiological processes and behaviour to regular changes in environmental conditions. The circadian clocks can measure time and optimise internal processes among different organs and with cycling environmental conditions. The circadian clocks are predominantly entrained by the photoperiod (the ratio of light (L) to dark (D) phases in the 24 h solar day), a very stable parameter for a given place on the Earth from year to year. High stability also qualifies the LD cycle as the best environmental cue also for other cycles, such as feeding, optimum time for reproduction and predator pressure.

The timing signal drives circadian oscillations over the body via the neuroendocrine and autonomic nervous systems to optimise physiological and behavioural processes and improve performance, health and survival [1]. All differentiated cells in the body contain clock genes, the expression of which is controlled via several interlocked transcriptional–translational feedback loops [2]. The understanding of this molecular mechanism was acknowledged by the Nobel Prize for Physiology or Medicine in 2017 [3]. The circadian clocks have reliably functioned (ticked) for millions of years and have been disturbed only during the last century by factors imposed on the environment by human activities. Among them, shift work was the first and, until now, probably is the most severe chronodisruptive condition, which has been intensively studied [4]. More recently, especially after the invention of highly effective light sources, such as the Light-Emitting Diode (LED), the negative effects of artificial light at night (ALAN) on human health and biodiversity have become highly prominent. It has been suggested that ALAN can interfere with the circadian control of physiological processes and behaviour, and the subsequent circadian disruption is recognised as being detrimental to health [5,6].

Currently, about 80% of people live under light-polluted skies [7], and light pollution increases by up to 10% per year [8], posing a serious threat to biodiversity and human health. Light pollution in cities substantially exceeds the level that can be experienced under a moonlight maximum (ca. 0.3 lx) [9] and can reach up to 150 lx [10]. Moreover, people can be exposed indoors to high levels of mistimed lighting from computer screens, TVs or safety lights, with blue light having the greatest detrimental effect [11]. This leads to chronodisruption of daily rhythms, such as sleep/activity cycles, rhythms in feeding and drinking, metabolism and the immune system, which are controlled by the neuroendocrine system.

In this featured review, we included publications on the effects of dim ALAN on clock-controlled hormonal outputs. We conducted a search on the PubMed/MEDLINE databases from October 2022 to March 2023, using the following search terms: ‘dim light at night’, ‘artificial light at night’, ‘circadian rhythm’, ‘chronodisruption’, ‘hormones’, ‘vasopressin’, ‘melatonin’, ‘glucocorticoids’, ‘corticosterone’, ‘reproductive hormones’, ‘testosterone’ and ‘thyroid hormones’. We characterised ‘dim light at night’ as exposure throughout an entire night, not exceeding an intensity of 10 lx. Relevant studies were assessed for inclusion by title and abstract, followed by a full-text review.

## 2. Circadian Control of the Neuroendocrine System

Circadian oscillations are prominent features of the neuroendocrine system, as the rhythmic production of hormones synchronises and coordinates body functions under rhythmic environmental conditions. Circadian rhythms are present in nearly all physiological systems and are imposed on the body by a hierarchically organised circadian system. At the top of the circadian system is the master pacemaker, which resides in the suprachiasmatic nuclei (SCN) of the hypothalamus. This paired structure is situated above the optic chiasma and consists of approximately 20,000 neurones in rats and 50,000 in humans [12]. The SCN receives inputs from the retina, providing information about the time of the solar day and synchronises the activity of the neurones in these hypothalamic nuclei. The central oscillator contains a heterogeneous population of neurons, differing in their neurochemical properties and connectivity [13]. Most neurons of the SCN form GABAergic synapses, but the specific expression of diverse neuropeptides gives them their identity [14].

In addition to the central circadian clock entrainable by light, several other brain structures receive a direct light input [15]. Moreover, various extra-SCN oscillators are described in the brain, among which the food-entrainable oscillator and the olfactory bulb could be the most important for the regulation of the circadian clock network [16]. These extra-SCN structures can be necessary for the control of behaviour but are understudied, and no data exist in relation to ALAN. In contrast to mammals, birds possess a multioscillatory circadian system. In addition to the SCN, the pineal gland and retina synthesise and release melatonin (MEL) in a circadian manner [17]. The chronodisruptive effects of ALAN on rhythms of physiology and behaviour are similar in birds and mammals, although only diurnal birds and mostly nocturnal mammals have been studied till now.

The SCN neurons control the major neuroendocrine systems through either a direct connection with neurosecretory neurons in the hypothalamic nuclei or intermediate connections. Neurons from the SCN project to the paraventricular nucleus (PVN) and the dorsomedial hypothalamic nucleus (DMH) to drive the circadian rhythmicity of the neuroendocrine system. Moreover, they innervate extra-hypothalamic structures, such as the organum vasculosum of the lamina terminalis (OVLT), to control the drinking behaviour [18,19]. It is possible that these specific neuronal clusters are differently sensitive to ALAN and can desynchronise the daily rhythms in different physiological systems through excitatory and inhibitory processes within the SCN and their respective targets.

An attenuated circadian control of the neuroendocrine system can weaken its adaptive value via the disruption of rhythms in circulating hormone levels. Despite this, only a few studies have explored the complete 24 h hormonal rhythms under dim ALAN exposure. Often, concentrations were measured only at one or two time points in 24 h, and such data may lead to misinterpretation of the underlying rhythmic changes because of possible shifts in acrophase or amplitude of different hormonal rhythms (Table 1).

The data presented in Table 1 demonstrate that only about one-third of the studies covered the whole 24 h cycle. Other studies measured the hormone levels only at one or two points during the day. Such an approach seems to have provided sufficient data on MEL suppression by ALAN but is insufficient for corticosterone, which also follows a distinct daily rhythm. Indeed, as shown in the Table, the studies differ in their findings, with increases, decreases, or no changes being reported. However, if the whole 24 h rhythm is considered, the studies reported diminished rhythmicity and a phase-advanced acrophase. Therefore, it is essential to perform measurements over the whole 24 h cycle and relate them to physiological and behavioural rhythms.

The SCN consists of two major regions, the ventromedial core and the dorsolateral shell. The core predominantly contains vasoactive intestinal peptide (VIP)-containing neurons, which receive a direct input from the retina. Efferent projections from these neurons transmit signals to vasopressinergic (AVP) neurons in the shell. Every cell in the SCN has internal clocks that must be coupled to ensure the proper synchronising function of the central clock [45,46]. The AVP-containing neurons are crucial for the regulation of inter-neuronal coupling, underlying a coherent circadian output of the SCN and the generation of robust circadian rhythms in behaviour [47]. Moreover, AVP is considered the major output of the SCN [48].

### Arginin-Vasopressin

It is generally accepted that the SCN temporally regulates physiology and behaviour, but the precise mechanisms are incompletely understood. In addition to the neural connections described above, the SCN can communicate circadian signals via diffusible humoral factors [49]. The main diffusible output signal can be AVP since it can cross the blood–brain barrier [50] in sensory circumventricular organs, including the OVLT, the subfornical organ and the area postrema [51]. Moreover, AVP in the cerebrospinal fluid exhibits circadian variation, with morning levels about five times higher than those at night in cats [52], and the SCN is suggested as the origin of this rhythmicity [53]. AVP synthesised in magnocellular neurones of the PVN and supraoptic nuclei (SON) and released into the circulation via the posterior pituitary is not under circadian control [52,54]. The PVN also contains smaller parvocellular neurones that co-secrete AVP and corticotrophin-releasing hormone (CRH) into the hypophyseal–portal bed and contribute to the regulation of adrenocorticotropic hormone (ACTH) release. Interestingly, the low-amplitude daily rhythm of *Avp* expression was found in the SON of adult male rats and was damped after ALAN exposure [32].

AVP acts via three types of receptors, V1a, V1b and V2, and plays a major role in the regulation of water and sodium homeostasis via its antidiuretic action on the kidneys, mediated by V2 receptors [55]. AVP secretion from the SON and PVN is stimulated by a rise in plasma osmolality and a decline in blood volume or stress [56]. Through V1 receptors expressed in vascular smooth muscle cells, AVP can control the blood supply to different organs and even metabolism in the liver [57]. The major mediator for the circadian function of AVP in the brain is the V1a receptor [58], because its activation during the subjective day was found to increase the amplitude of the firing rates of SCN neurons [59]. Thus, it is generally accepted that V1a signalling has an important role in the generation of overt circadian rhythms [60]. Mice genetically lacking V1a and V1b receptors are resistant to jet lag [61] and show dampening of rhythmic clock gene expression in response to the advanced LD cycle [62]. Interestingly, a decrease in the AVP-immunoreactive neurons in the SCN was recorded in patients with type 2 diabetes, exhibiting circadian misalignment [63].

Recent chromogenic and optogenetic studies in mice have shown that AVP-containing SCN neurons projecting to the OVLT ensure the circadian control of anticipatory thirst before sleep [19]. This pronounced increase in water intake during the last part of the active period, which protects against dehydration in the absence of drinking during sleep, can be compromised by exposure to dim ALAN [32]. ALAN changed the daily pattern of the behavioural rhythms in food and water intake and profoundly suppressed the clock-controlled surge of drinking in male rats two hours prior to the onset of the light period. These effects were associated with attenuated rhythms in *Avp* expression in the SCN and the elimination of vasopressin rhythmicity in the general circulation. The same dim ALAN conditions changed the daily rhythms of physical activity and metabolism [64]. The *Avp* expression is transcriptionally regulated by clock genes via E-box control elements [65], and the vasopressinergic SCN neurons can control the major neuroendocrine axes through either direct connections with neuroendocrine neurons in the PVN or interneurons in the subparaventricular zone (subPVN) and the DMH [48]. In this way, the disturbed circadian output of the SCN in ALAN-exposed rats can desynchronise the circadian rhythms in the neuroendocrine system.

A recent study indicates that specific AVP-containing neurones in the SCN are capable of not only sustaining specific rhythmic outputs but also integrating external and internal non-photic signals and producing circadian time-specific adaptive responses [66]. This possibility was demonstrated in response to dehydration, but the same mechanism can be involved in the timing of core temperature or preovulatory luteinizing hormone (LH) peaks in female rodents [67].

**Figure 1:**
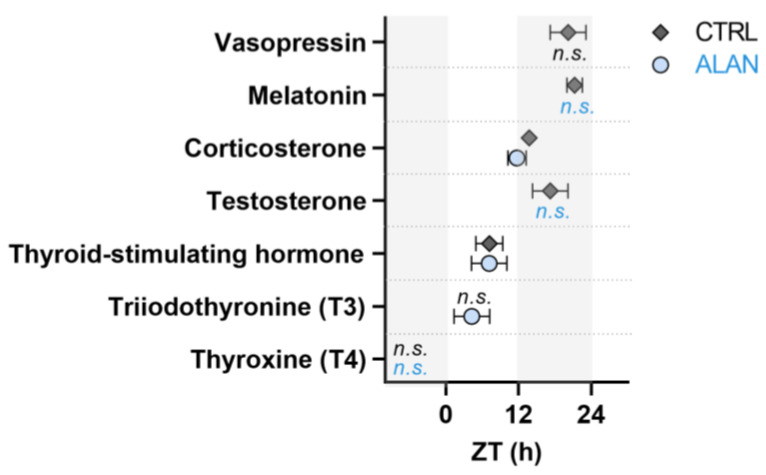
Effects of dim ALAN on the acrophases of plasma hormones. Rats were either kept under control conditions (CTRL, grey, 12L:12D) or exposed to dim ALAN (blue, 12L:12DL) the entire night for two weeks. *n.s.* = not significant rhythm. ZT = Zeitgeber time. Data are presented as mean ± SEM. The Figure was created based on the results from [32].

Desynchronised or mistimed daily hormonal rhythms can underlie and determine the chronodisruption of physiological processes and behaviour and can participate in the development of different civilisation diseases. However, the complete 24 h rhythms in different hormonal axes have been insufficiently investigated. A recent study [32] explored the complete 24 h rhythms of several end-point hormones of the main neuroendocrine axes in mature rats exposed to dim ALAN during the whole night (Figure 1). The study showed that the circadian rhythms of important hormones, such as MEL, testosterone and vasopressin, were eliminated, and the corticosterone rhythm was phase-advanced. The rhythm of thyroid-stimulating hormone (TSH) was not affected by dim ALAN, and surprisingly, the triiodothyronine (T_3_) plasma concentrations exhibited daily rhythmicity after low levels of ALAN.

In the next sections, we will summarise the changes in the daily rhythms in major endocrine axes, which can be affected by light pollution, with possible negative effects on physiology and behaviour.

## 3. Melatonin

Melatonin is the endocrine output of the SCN that transmits circadian information through the whole body and stabilises the circadian rhythms. Its receptors are present in most organs, including the SCN, and MEL biosynthesis is immediately inhibited by light. Melatonin concentrations exhibit distinct daily rhythms, reaching a maximum during the night in both nocturnal and diurnal animals. The duration of high MEL levels corresponds with the length of the dark period, and therefore, the daily rhythms of this hormone can serve not only as clocks but also as a calendar. Melatonin is well known for its pleiotropic effects [68], making suppressed night-time MEL levels a good candidate for explaining the negative consequences of ALAN on human well-being and health. However, it is necessary to mention that MEL effects were usually measured after the administration of supraphysiological or pharmacological doses (mg/kg of body weight), and it has not been proven that physiological concentrations (pg/mL) elicit the same effects. Therefore, more studies with physiological MEL doses applied during dim-light nights to compensate for the suppressed endogenous MEL production are needed.

### 3.1. Melatonin and ALAN

Melatonin is the best-studied hormone in the ALAN context in different systematic classes of animals [69] and has also been reviewed [70]. The nocturnal suppression of MEL is generally considered the best marker of light pollution because its concentrations can be rather easily measured in all biological fluids, not only in plasma and serum, but also noninvasively in saliva or urine. Surprisingly, a very high interindividual variability in MEL response to ALAN was recently documented in healthy humans [71]. An illuminance of 6 lx effectively decreased the plasma MEL levels in sensitive probands, whereas in the most resistant man, an illuminance of around 350 lx was needed. Moreover, in a real-life study, the whole night exposure to 5 lx of LED light decreased urinary 6-sulfatoxymelatonin in adult humans, and levels as low as 1 lx interfered with the quality of sleep when evaluated on an individual basis [72]. Thus, the huge interindividual variability complicates a group comparison, may underestimate the suppressive effects of ALAN on MEL and must be considered in statistical evaluation. Still, it is not clear which factors underlie this extensive interindividual variability. In addition to the genetic background, epigenetic and developmental aspects, including personal history, are also possible and should be studied. In laboratory animals, such interindividual variability has not yet been demonstrated and should be evaluated. On the other hand, several strains of mice do not synthesise MEL and exhibit distinct circadian rhythms in physiology and behaviour [73].

### 3.2. Melatonin in Diurnal and Nocturnal Species

Melatonin suppression by ALAN was documented extensively in nocturnal and diurnal species, and mainly birds were investigated in the latter group (Table 1). In nocturnal rodents, an illuminance at the level of full-moon nights (0.2 lx) significantly decreased MEL concentration in the circulation [28]. It is expected that nocturnal animals are more sensitive to ALAN than diurnal animals, but for example, also in diurnal Zebra finches, light of 1.5 lx significantly decreased the MEL levels [37]. A similar sensitivity was reported for other birds, such as Indian house crows (*Corvus splendens*) [39] and great tits [43], and can be considered typical for avian species. However, the direct comparison of the two systematic groups is limited by the fact that MEL suppression has been investigated mainly in nocturnal mammals and in diurnal birds.

### 3.3. Melatonin during Development

Maternal MEL can pass through the placenta into the foetal circulation [74] and serve as a hormonal signal for the foetus, as MEL receptors are found in different foetal organs, including the SCN [75]. ALAN exposure during pregnancy or the lactating period can impact the development of MEL rhythms during early ontogeny, imposing potential long-lasting effects on the sensitivity of the circadian system in adulthood. A recent study showed that the development of the plasma MEL rhythm was delayed in offspring born to female rats exposed to ALAN during pregnancy [76]. The possible importance of MEL during pregnancy is illustrated by the fact that its levels in pregnant women substantially increase from gestational week 32 and are normalised only after delivery [77]. Similarly, in rats, the night-time MEL levels rise during the third week of pregnancy, peak right before parturition and return to the non-pregnant values by postpartum day 2 [78]. However, the data on the MEL profile in pregnant females under disturbed lighting conditions are limited, indicating a serious gap that should be eliminated in future studies.

The administration of MEL to pregnant female rats with suppressed endogenous MEL production entrained the clock gene expression profile in the offspring SCN. Interestingly, the same treatment failed to affect clock gene expression in the liver [79], suggesting that the central but not the hepatic oscillators in the foetus are sensitive to this hormone. In another model, chronic shifts of the photoperiod during pregnancy altered the circadian organisation of pregnant diurnal capuchin monkey females, but their MEL rhythm was preserved, although with a lower amplitude. Surprisingly, in adult male offspring exposed to shifts prenatally, the MEL rhythm was eliminated, whereas it was present in control animals [80].

## 4. The Hypothalamic–Pituitary–Adrenal (HPA) Axis

After MEL, glucocorticoids are the second most investigated hormones in response to ALAN. They are released from the adrenal cortex and exhibit a distinct circadian rhythm with a rise around the onset of the active phase in both diurnal and nocturnal species. The central clock regulates glucocorticoid production via (1) the HPA axis and (2) the autonomic nervous system [48]. Vasopressinergic neurons from the SCN project to the subPVN and DMH and either excite or inhibit CRH-containing neurons in the PVN, depending on the diurnality/nocturnality of animals. In nocturnal rats, during the light period, the CRH-containing neurons are inhibited via GABAergic neurons from the subPVN and DMH. On the other hand, in diurnal animals, the vasopressinergic SCN neurons act on excitatory glutamatergic projections in the subPVN and DMH, stimulating the CRH-containing neurons in the PVN [48,81]. Besides the SCN outputs, the functional clock in the CRH neurons of the PVN is also important to drive the circadian pattern of corticosterone release [82]. The CRH neurons project into the median eminence, where CRH is released into the portal system and stimulates the release of ACTH from the anterior pituitary gland in a circadian manner. In the adrenal cortex, ACTH promotes the rhythmic production and release of glucocorticoids. The second mechanism contributing to the circadian rhythms of glucocorticoid levels is the autonomic nervous system, which affects the cell sensitivity of the adrenal cortex to ACTH [83].

Cortisol is a major glucocorticoid in humans, and corticosterone represents a dominant glucocorticoid in rats and mice. Glucocorticoids exert a wide spectrum of effects, since their receptors are detected in almost all tissues, except the SCN [84,85]. Moreover, the glucocorticoid-responsive element is present in the regulatory areas of many genes, including clock genes, mediating the entrainment of the peripheral clocks in the liver and synchronising the large part of the hepatic transcriptome [86]. This way, glucocorticoids are involved in the control of metabolism, securing a sufficient energy supply via lipolysis, a decreased glucose uptake in muscle and adipose cells and elevated gluconeogenesis in the liver [87,88,89,90].

Disruptive effects of ALAN on plasma glucocorticoid levels have been reported in several studies, but usually, the hormone levels were measured only at one or two time points over a 24 h cycle [91]. This has led to some contradictory findings [24,28,64]. In our recent paper, we showed that rats exposed to dim ALAN (2 lx) for two weeks preserved corticosterone rhythmicity, but the rhythm was phase-advanced and had a lower amplitude. The shift of the acrophase to earlier hours indicated a chronodisruption of glucocorticoid rhythmicity, which spontaneously peaked at the beginning of the active phase in both nocturnal and diurnal animals. If an earlier rise in cortisol occurs in people under ALAN conditions, it can result in earlier awakening and interfere with sleep quality in stressed individuals. In nocturnal animals, the shift can phase-advance their physical activity to the twilight hours, with increased predator pressure. Thus, we emphasise the need to analyse the complete 24 h rhythms of physiological variables after exposure to ALAN. The reduced amplitude of the corticosterone rhythm after ALAN is rather surprising because an increase is usually expected. However, this finding presents new challenges because lower glucocorticoid levels are often associated with autoimmune diseases [92]. This is a novel field for ALAN research, which requires further attention because of the increasing incidence of autoimmune diseases in countries with a higher level of light pollution.

## 5. The Hypothalamic–Pituitary–Gonadal (HPG) Axis

The SCN confers circadian information to gonadotropin-releasing hormone (GnRH) neurons through several converging direct and indirect pathways, which control the HPG axis. The GnRH release in the median eminence subsequently orchestrates the production of gonadotropins, LH and follicle-stimulating hormone. They exhibit significant daily rhythms with a peak at the beginning of the active phase, 12 h apart in diurnal and nocturnal species, respectively. Because the LH surge is gated by the SCN, the system can be prone to circadian disruption, which has been more extensively studied in females than in males [93]. The predominant research on chronodisruption in females is motivated by the reproductive problems of women involved in shift work [94]. In men, such problems have not been sufficiently studied till now. Interestingly, *Bmal1* knockout mice are infertile, although male mice have only mild HPG axis impairments. They do not engage in mating behaviour, probably due to neural circuitry defects in transforming the olfactory cues necessary for the mating behaviour [95].

Surprisingly, very limited information exists about disturbances in the daily rhythms of reproductive hormones because of light pollution. A recent study [32] showed the elimination of the plasma testosterone daily rhythm in male Wistar rats exposed to environmentally realistic ALAN levels (2 lx) for the entire night. It is possible to expect that the eliminated rhythm of this dominant androgen can have negative consequences on the timing of sex behaviour, and future studies are needed in this field.

Since the SCN clock is entrained by the LD cycle, it is conceivable that a disturbed LD signal may affect the photoperiodic control of the HPG axis and the coordination of delicate physiological and behavioural processes, which are required for a successful seasonal reproduction. Indeed, several studies have shown that exposure to ALAN accelerates the photoperiodically driven reproductive development [96,97]. More studies with various species in different environmental situations are needed to show if this acceleration has positive or negative consequences because of a possible dissonance with periodic seasonal conditions.

## 6. The Hypothalamic–Pituitary–Thyroidal (HPT) Axis

Thyroid hormones have a key role in the regulation of metabolism and development. The HPT axis is under a strong circadian control [98] because, in the hypothalamus, SCN fibres innervate neurons expressing thyrotropin-releasing hormone (TRH), which in the anterior pituitary gland controls the rhythmic production of TSH. In the thyroid gland, TSH regulates the synthesis and release of T_3_ and thyroxine (T_4_), which bind to their nuclear receptors, found in nearly all organs and tissues, except for the retina and testes [99]. Both TRH and TSH exhibit distinct circadian rhythms, with higher levels during the inactive phase [100], and are in antiphase in nocturnal and diurnal species [101], similar to what observed for the HPA axis. It is hypothesised that both axes are controlled similarly, with a critical role for AVP [102]. In both, the autonomic nervous system can play an important role because retrograde tracing experiments revealed multisynaptic connections between the SCN and the adrenal and thyroid glands [103,104].

In contrast to the distinct rhythms for TSH, only low-amplitude rhythms or arrhythmicity have been reported for peripheral T_3_ and T_4_, suggesting that other circulating hormones and peripheral deiodination can modulate the HPT axis activity. Peripheral circadian clocks have been identified in the thyroid gland of rats [105] and human primary thyrocytes [106]. Their role in the control of the circadian rhythms of thyroid hormones is probably less important because, in hypophysectomised rats, the circadian thyroid hormone rhythms were eliminated, but the expression of circadian clock genes in the thyroid was unaffected [105]. Surprisingly, little is known about the consequences of ALAN on rhythms in the HPT axis [32,107].

## 7. Conclusions

Circadian disruption is recognised as being detrimental for biodiversity and health because it interferes with physiological and behavioural rhythms, which are controlled by the neuroendocrine system. The presented data outline the diminished central clock function of the SCN after exposure to low levels of ALAN. The weakened circadian output from the SCN results in eliminated daily rhythms of MEL, testosterone and vasopressin and disrupted rhythm of corticosterone. Further research is needed to elucidate possible negative consequences on physiology and behaviour and design effective mitigation strategies to eliminate or minimise the effects of ALAN on human health and biodiversity in natural ecosystems.

## Figures and Tables

**Table 1:** Effects of dim artificial light at night (ALAN) on hormones. AVP = arginine vasopressin. CORT = corticosterone. CTRL = control group. DL = dim light phase. F = female. GnIH = gonadotropin-inhibiting hormone. GnRH = gonadotropin-releasing hormone. L = light phase. lm = lumen. M = male. MEL = melatonin. *Mel1* = melatonin receptor 1. SHR = spontaneously hypertensive rats. T_3_ = triiodothyronine. T_4_ = thyroxine. TESTO = testosterone. TSH = thyroid-stimulating hormone. *Tshr* = TSH receptor. ZT = zeitgeber time.

**Species**	**Light Conditions**	**Hormone**	**Effect**	**Sampling Time**	**Ref.**
Swiss Webster mice 8 weeks old M	16L:8DL L = 150 lx DL = 5 lx (8 weeks)	CORT	Unaffected (ZT7, ZT15) Unaffected (rhythm)	2 time points (ZT7, ZT15) 6 time points in 4 h intervals over 24 h	[20]
Swiss Webster mice 8 weeks old M	14L:10DL L = 150 lx DL = 5 lx (24 h)	CORT	Unaffected (sham-operated)	1 time point (midday)	[21]
C57BL/6J mice 3 weeks old M	12L:12DL L = 150 lx DL = 5 lx (3 weeks)	CORT	↑	1 time point(unknown time)	[22]
C57BL/6J mice 8 weeks old M	12L:12DL L = 150 lx DL = 5 lx(4 weeks)	CORT	↑	1 time point (ZT0)	[23]
Siberian hamsters Adult F	16L:8DL L = 150 lx DL = 5 lx (8 weeks)	CORT	Suppressed amplitude (lower at ZT15)	6 time points in 4 h intervals over 24 h	[24]
Siberian hamsters 93–114 days M (reproductive)	8L:16DL L = 150 lx DL = 5 lx (8 weeks)	*Mel1* *Tshr* *GnRH* *GnIH* (in hypothalamus and pars tuberalis)	↑ ↑ ↑ ↑	1 time point (ZT8)	[25]
Grass rats 10 weeks old M	14L:10DL L = 150 lx DL = 5 lx (3 weeks)	CORT	↑	1 time point (ZT6)	[26]
Sprague–Dawley rats 35–50 g M	12L:12DL L = 300 lx DL = 0.2 lx (6 weeks)	MEL CORT	↓ Nocturnal levels (ZT22) Phase-advanced	2 time points (ZT10, ZT22) 6 time points in 4 h intervals over 24 h	[27]
Sprague–Dawley rats 3–4 weeks old M	12L:12DL L = 300 lx DL = <10 lx (5 weeks) Red light	MEL CORT	Suppressed amplitude Phase-advanced, suppressed amplitude	6 time points in 4 h intervals over 24 h	[28]
Ovariectomized, athymic, inbred nude rats 1–2 weeks old F	12L:12DL L = 300 lx DL = 0.2 lx (6 weeks)	MEL	Suppressed amplitude	6 time points in 4 h intervals over 24 h	[29]
Wistar rats 18 weeks old M	12L:12DL L = 150 lx DL = 2 lx (2 or 5 weeks)	MEL	↓ Nocturnal levels (ZT21)	2 time points (ZT9, ZT21)	[30]
Wistar rats 18 weeks old M	12L:12DL L = 150 lx DL = 2 lx (2 or 5 weeks)	CORT	↑	1 time point (ZT3-6)	[31]
Wistar rats 275 ± 3 g M	12L:12DL L = 150 lx DL = 2 lx (2 weeks)	MEL (pineal) MEL (plasma) CORT TESTO AVP TSH T_4_ T_3_ T_3_/T_4_ ratio	Suppressed amplitude Eliminated rhythm Phase-advanced, suppressed amplitude Eliminated rhythm Eliminated rhythm Unaffected Unaffected Gained rhythm Unaffected	6 time points in 4 h intervals over 24 h	[32]
Wistar rats 7 weeks old F	12L:12DL L = 250 lx DL = 5–7 lx (5 weeks)	MEL CORT	↓ Nocturnal levels (ZT14) Unaffected	2 time points (ZT2, ZT14)	[33]
Zebra finches <1 year old M, F	10L:14DL L = 95 lm DL = 1.5 lx (10 nights)	MEL	Unaffected Atypical rhythm in CTRL	6 time points in 4 h intervals over 24 h	[34]
Zebra finches Adult M, F	12L:12DL L = 400 ± 50 lx DL = 3 ± 1 lx (10 days) Blue light	MEL CORT	Eliminated rhythm Eliminated rhythm	6 time points in 4 h intervals over 24 h	[35]
Zebra finches Adult F	12L:12DL L = 150 lx DL = 5 lx (3 weeks)	MEL T_4_	↓ Nocturnal levels (midnight)↓ Daytime levels (midday)	2 time points (midday, midnight)	[36]
Zebra finches Adult F	14L:10DL L = 1200 lx DL = 0.5, 1.5, 5 lx (3 weeks)	MEL	↓ Nocturnal levels (1.5 and 5 lx)	2 time points (midday, midnight)	[37,38]
Indian house crows Adult	12L:12DL L = 150 lx DL = 6 lx (10 days)	MEL CORT	↓ Nocturnal levels (ZT18) Unaffected	2 time points (ZT6, ZT18)	[39]
Indian house crows Adult	12L:12DL L = 150 lx DL = 6 lx (10 days)	MEL	↓ Nocturnal levels (ZT18)	2 time points (ZT6, ZT18)	[40]
Tree sparrows Adult M	Urban area Rural area	MEL	↓ Nocturnal levels, suppressed amplitude	6 time points in 4 h intervals over 24 h	[41]
European blackbirds Adult M	L = 250–1250 lx DL = 0.3 lx D = 0.0001 lx (CTRL) Photoperiod followed the local natural day length	MEL	↓ Nocturnal levels, suppressed amplitude	4 time points (winter: 6:00, 12:00, 18:00, 24:00, summer: 3:00, 12:00, 21:00, 24:00)	[42]
Great tits 1–4 years oldM	8.25L:15.75DL L = 1000 lx DL = 0.05, 0.15, 0.5, 1.5, 5 lx (4 weeks)	MEL	↓ Nocturnal levels (with increasing intensity)	3 time points (morning, midday, midnight)	[43]
Great tits Adult M,F	L = natural light intensity DL = 8.2 ± 0.3 lx (10–12 days) Photoperiod followed the local natural day lengthField	CORT	↑ (under white light)	1 time point (9:00–15:00)	[44]
